# Validation of the Gait Disorientation Test in children with concussion

**DOI:** 10.3389/fped.2022.927708

**Published:** 2022-08-22

**Authors:** Abdulaziz A. Alkathiry, Saud F. Alsubaie, Bara A. Alsalaheen, Susan L. Whitney

**Affiliations:** ^1^Department of Physical Therapy and Health Rehabilitation, Majmaah University, Majmaah, Saudi Arabia; ^2^Department of Physical Therapy and Health Rehabilitation, College of Applied Medical Sciences in Al-Kharj, Prince Sattam bin Abdulaziz University, Al-Kharj, Saudi Arabia; ^3^Department of Physical Therapy, College of Health Sciences, University of Michigan-Flint, Flint, MI, United States; ^4^Department of Physical Therapy, School of Health and Rehabilitation Sciences, University of Pittsburgh, Pittsburgh, PA, United States

**Keywords:** Gait Disorientation Test, concussion, children, gait speed, balance, mild TBI (mTBI)

## Abstract

**Background:**

Mild traumatic brain injury (mTBI) or concussion is a complex injury that is difficult to diagnose and assess. There are negative impacts on cognition, balance, and mobility after a concussion. The Gait Disorientation Test (GDT) is an objective measure that assesses a person's balance ability by comparing the walking time with eyes open and the walking time with eyes closed in a standardized walking task. The purpose of this study was to assess the validity and the diagnostic properties of the GDT in children with concussions.

**Methods:**

Thirty-six children with concussions, and 91 controls aged between 9 and 18 years old participated in the study. Participants completed demographics, the GDT, the Functional Gait Assessment (FGA), the Pediatric Vestibular Symptom Questionnaire (PVSQ), and the Pediatric Visually Induced Dizziness Questionnaire (PVID).

**Results:**

Children with concussions showed higher (worse) GDT scores (*M* = 2.18 ± 1.93 s) than healthy controls (*M* = 1.13 ± 0.95 s), which was statistically significant (*P* = 0.014).

**Conclusion:**

The GDT was able to distinguish between children with concussions and healthy controls. Given the simplicity of the GDT, it can be used to assist in discriminating between children with and without concussion.

## Introduction

A concussion is a common brain injury, which may lead to multiple health impairments including physical and cognitive symptoms, such as headache, dizziness, balance impairments, and visual problems (1, 2). In the last decade, concussion awareness has increased in the medical community and has largely focused on adult injuries (3). Several studies indicated that adolescents are more likely to develop concussions compared to adults (4, 5). Previous studies have found that concussion injuries have a negative impact on cognition, balance, and mobility, which recover at different rates post-concussion (6, 7). Utilizing a functional performance test, as a part of a comprehensive examination of a concussion, may enable health clinicians to better determine the trajectory of recovery following a concussion (8–11). A recent study suggested a new measure, the Gait Disorientation Test (GDT), which is the difference in the time needed to finish a 20-feet walking task between performing the task with eyes open and eyes closed (12). The GDT has been shown to possess excellent discriminative ability to distinguish between adults with vestibular impairments and normal adults.

The purpose of this study was to assess the validity and the diagnostic properties of the GDT in children with and without concussions.

## Methodology

Thirty-six children with concussion/mTBI and 91 controls between 9 and 18 years of age participated in the study. Participants with concussions who were seeking medical attention for their concussion were recruited after a neurologic and neuro-otologic examination from a tertiary balance center at the University of Pittsburgh Medical Center (UPMC), Pittsburgh, PA, USA. Controls were recruited from middle and high schools in Pittsburgh, PA, USA. Informed consent was obtained from all participants and their guardians. The study was approved by the institutional review board from the University of Pittsburgh and was conducted in accordance with the Declaration of Helsinki.

Participants from both groups completed demographics and the Functional Gait Assessment (FGA), which is a performance-based test that include 10 walking and stair-climbing tasks (13). The performance of each task is rated by the test administrator from 0 to 3 using specific criteria for each score. The FGA total score is calculated by adding the scores of all the tasks and ranged from 0 to 30, with higher scores indicating better performance. The children with concussions completed the Pediatric Vestibular Symptom Questionnaire (PVSQ), which is a self-reported questionnaire comprising 11 questions about the frequency of vestibular symptoms during the past month using a 4-point Likert scale that ranged from “never” to “most of the time”(14). The total score of PVSQ ranged from 0 to 3 with a higher score indicating worse symptoms. The PVSQ score is a normalized score that is calculated by adding all scores from the answered questions divided by the number of questions answered. The cut-off score of the PVSQ to discriminate between healthy children and children with vestibular impairments was found to be ≥0.68 (14).

The Pediatric Visually Induced Dizziness Questionnaire (PVID) is a self-reported questionnaire comprising 11 questions about the frequency of feeling dizzy and unsteady in different places and situations during the past month using a 4-point Likert scale that ranged from “never” to “most of the time”(15). The total score of the PVID ranged from 0 to 33 with higher scores indicating worse symptoms. The PVID score is normalized by dividing the total score by the number of questions answered. The cut-off score of the PVID to discriminate between healthy children and children with vestibular impairments was found to be ≥0.45 (15).

The GDT is measured in seconds and was calculated by subtracting the time needed to complete the 10-m gait speed test with eyes closed (GS-EC) and the time needed to complete the normal 10-m gait speed test with eyes open (GS). Both GS and GS-EC were timed during the FGA tasks similar to Grove et al. (12, 13). All investigators participated in the data collection of healthy participants. The main investigator performed all the testing and recruitment for the participants with concussions.

### Statistical analysis

Outcomes were tested using the Shapiro–Wilk test of normality of the distribution to determine the appropriate statistical methods. Descriptive data were reported with the appropriate statistical methods using means and standard deviations, median and interquartile ranges, or frequency and percentages. Comparisons between children with and without concussion were performed using independent sample *t*-tests or the Man–Whitney *U*-test. Group comparisons were further examined using a one-way ANCOVA to adjust for age, gender, and height differences. The GDT, GS, and GS-EC were tested, as appropriate, using Person or Spearman correlation coefficients against the FGA, PVSQ, and PVID to assess their concurrent validity. The receiver operator characteristic (ROC) analysis was used to assess the diagnostic ability of GDT, GS, and GS-EC to discriminate between the concussed and control children. The optimal cut-off values were calculated using Youden's Index (16). The cut-off values were used to produce the contingency tables for the GDT, GS, and GS-EC. The contingency tables were used to calculate the specificity, the sensitivity, the Positive Likelihood Ratio (LR+) and Negative Likelihood Ratio (LR–), and the Diagnostic Odds Ratio (DOR). Statistical analysis was performed using SPSS. Youden's Index, specificity, sensitivity, LR+, LR–, and DOR were calculated using Microsoft Excel.

## Results

Thirty-six children with concussion/mTBI aged between 9 and 17 years old (*m* = 14.2, SD = 2.4 years) and 91 healthy children aged between 14 and 18 years old (*m* = 15.6, SD = 1.1 years) completed the study. Significant differences between groups were found in age, weight, and height (*P* < 0.01; Table 1).

**Table 1:** Characteristics of healthy and concussed children.

	**Concussion *n* = 36**	**Healthy *n* = 91**	***P*-value**
	***M* (SD)**	***M* (SD)**	
Age^∧^	14.22 (2.42)	15.62 (1.05)	0.009^**^
Gender (Male, %)^#^	12 (33%)	47 (52%)	0.062
Weight (Kg)^∧^	55.66 (17.95)	63.41 (14.66)	0.002^**^
Height (cm)	159.81 (13.76)	169.26 (8.43)	<0.001^**^
BMI^∧^	21.29 (4.00)	22.06 (4.49)	0.409

∧ *Mann-Whitney U-test*.

# *Chi square*.

** *P < 0.01*.

*BMI, Body Mass Index*.

Children with concussion were recruited 4–434 days after injury [interquartile range (IQR) = 115 days; *m* = 130.1; SD = 144.5 days]. Twenty-four (67%) children with concussions reported having symptoms of dizziness, 17 children reported spinning sensation (47%), and 17 children reported migraine (47%). Thirty-four (94%) children with concussion tested positive on the PVSQ [*m* = 1.21, SD = 0.44] or the PVID [*m* = 1.38, SD = 0.81] tests using the cut-off scores reported for children with concussion (14, 15). Children with concussions reporting dizziness demonstrated significantly worst performance on GS, GS-EC, GDT, and FGA than those without dizziness (Table 2).

**Table 2:** A comparison of the GDT, GS, GS-EC, and FGA in children with concussion.

	**Dizziness**			**Spinning**			**Migraine**		
	**Yes (*n* = 24)**	**No (*n* = 12)**	***T*-test**	**Yes (*n* = 17)**	**No (*n* = 19)**	***T*-test**	**Yes (*n* = 17)**	**No (*n* = 19)**	***T*-test**
GDT (s)	2.55 (2.22)	1.45 (0.88)	0.044^*^	2.32 (1.62)	2.06 (2.22)	0.686	2.15 (2.24)	2.21 (1.68)	0.931
GS (m/s)	1.09 (0.14)	1.27 (0.12)	<0.001^**^	1.12 (0.15)	1.18 (0.17)	0.316	1.12 (0.14)	1.18 (0.17)	0.234
GS-EC (m/s)	0.79 (0.21)	0.98 (0.12)	0.007^**^	0.82 (0.20)	0.89 (0.21)	0.324	0.83 (0.18)	0.87 (0.22)	0.582
FGA	26.83 (2.09)	28.58 (0.90)	0.002^**^	27.06 (2.56)	27.74 (1.56)	0.338	27.41 (1.78)	27.42 (2.39)	0.990

* *P < 0.05*.

** *P < 0.01. GDT, Gait Disorientation Test; GS, Gait Speed; GS-EC, Gait Speed with Eyes Closed; FGA, Functional Gait Assessment (optimal score is 30)*.

Significant differences in the GDT score were found between children with concussion (*M* = 2.18 ± 1.93 s) and healthy controls (*M* = 1.13 ± 0.95 s; *P* =0.014), indicating that children with concussion demonstrated larger changes compared to healthy controls in walking speed when walking with eye closed compared to eyes open. Gait speed did not differ between the groups (*P* = 0.108), while gait speed with eyes closed was significantly slower in children with concussions than healthy controls (*P* < 0.001). The FGA demonstrated a statistically significant difference between groups demonstrating better performance by the healthy controls (*P* = 0.003). Results from One-way ANCOVA showed that the GDT, the GS-EC, and the FGA were significantly different between the groups with better performance in the control group compared to the concussion group (Table 3).

**Table 3:** A comparison of the GDT, GS, GS-EC and FGA in children with concussion and healthy controls.

	**Concussion *n* = 36**	**Healthy *n* = 91**	***T*-test**	**ANCOVA^#^**
GDT (s)	2.18 (1.93)	1.13 (0.95)	0.014^*^^∧^	0.005^**^
GS (m/s)	1.15 (0.16)	1.20 (0.16)	0.108	0.078
GS-EC (m/s)	0.85 (0.20)	1.00 (0.17)	<0.001^**^	0.001^**^
FGA	27.42 (2.09)	28.52 (1.39)	0.003^**^^∧^	0.020^*^

# *Adjusted for age, height, and gender*.

∧ *Mann-Whitney U-test*.

* *P < 0.05*.

** *P < 0.01*.

*GDT, Gait Disorientation Test; GS, Gait Speed; GS-EC, Gait Speed with Eyes Closed; FGA, Functional Gait Assessment (optimal score is 30)*.

One-way ANCOVA was conducted to determine a statistically significant difference between children with and without concussion on GDT, GS, GS-EC, and FGA controlling for age, height, and gender. There was a significant effect of the group after controlling for age, height, and gender on GDT, *F*_(1,122)_ = 8.305, *P* = 0.005; GS-EC, *F*_(1,122)_ = 11.201, *P* = 0.001; and FGA, *F*_(1,122)_ = 5.600, *P* = 0.020. There was no significant effect of group on GS after controlling for age, height, and gender, *F*_(1,122)_ = 3.164, *P* = 0.078 (Table 3).

The GDT, gait speed with eyes closed, and the FGA demonstrated significant differences between the groups and were further analyzed for their discriminant validity using ROC analyses. The optimal cut-off scores for the GDT, gait speed with eyes closed, and FGA were determined using the Youden's Index and were 1.5 s,0.9 m/s, and 28 points, respectively (Table 4). For a GDT threshold of 1.5 s, we found that the sensitivity and specificity were.58 and.77, respectively. The diagnostic odds ratio (DOR) = 4.67, LR+ = 2.53, and LR– = 0.54. Sensitivity, specificity, DOR, and positive and negative LRs are reported for gait speed with eyes closed and the FGA in Table 5.

**Table 4:** Summary of ROC analyses.

**Test**	**Threshold^#^**	**AUC (95% CI)**	**SE**	***P*-value**
GDT (s)	1.5	0.682 (0.574–0.790)	0.055	0.001^**^
GS (m/s)	1.3	0.594 (0.486–0.703)	0.056	0.089
GS-EC (m/s)	0.9	0.698 (0.592–0.804)	0.054	<0.001^**^
FGA	28	0.668 (0.563–0.772)	0.053	0.002^**^

# *Youden's Index*.

* *P < 0.05*.

** *P < 0.01*.

*AUC, area under the curve; SE, standard error; GDT, Gait Disorientation Test; GS, Gait Speed; GS-EC, Gait Speed with Eyes Closed; FGA, Functional Gait Assessment*.

**Table 5:** Diagnostic performance.

	**TP**	**FP**	**FN**	**TN**	**Sn (95% CI)**	**Sp (95% CI)**	**LR+ (95% CI)**	**LR- (95% CI)**	**DOR (95% CI)**
GDT	21	21	15	70	0.58 (0.42–0.74)	0.77 (0.68–0.86)	2.53 (1.59–4.03)	0.54 (0.36–0.81)	4.67 (2.05–10.63)
GS	30	59	6	32	0.83 (0.71–0.96)	0.35 (0.25–0.45)	1.29 (1.05–1.59)	0.47 (0.22–1.03)	2.71 (1.02–7.20)
GS-EC	23	24	13	67	0.64 (0.48–0.80)	0.74 (0.65–0.83)	2.42 (1.59–3.69)	0.49 (0.31–0.77)	4.94 (2.17–11.27)
FGA	24	37	12	54	0.67 (0.51–0.82)	0.59 (0.49–0.69)	1.64 (1.17–2.30)	0.56 (0.34–0.92)	2.92 (1.30–6.56)

*TP, true positive; FP, false positive; FN, false negative; TN, true negative; Sn, sensitivity; Sp, specificity; LR+, positive likelihood ratio; LR-, negative likelihood ratio; DOR, diagnostic odds ratio; CI, confidence interval; GDT, Gait Disorientation Test; GS, gait speed; GS-EC, gait speed with eyes closed; FGA, functional gait assessment*.

In children with concussion, the GDT significantly correlated with the FGA demonstrating better outcomes with a decreased or smaller GDT (*P* < 0.01). Gait speed significantly correlated with the FGA, PVSQ, and PVID demonstrating better outcomes with increased gait speed (*P* < 0.05). Gait speed with eyes closed significantly correlated with the FGA and the PVID demonstrating better outcomes with faster gait speed while walking with eyes closed (*P* < 0.01). Time since injury did not show a significant correlation with the functional tests GS, GS-EC, GDT, and FGA or the questionnaires PVSQ and PVID (Table 6).

**Table 6:** Correlation between the dependent variables for children with concussion/mTBI.

	**Onset**	**GDT**	**GS-EC**	**GS**
Onset		−0.281	0.187	0.148
FGA	0.215	−0.434^**^	0.686^**^	0.729^**^
PVSQ	−0.186	0.247	−0.319	−0.345^*^
PVID	0.072	0.354^*^	−0.459^**^	−0.345^*^

* *P < 0.05*.

** *P < 0.01*.

*GDT, Gait Disorientation Test; GS, gait speed; GS-EC, gait speed with eyes closed; FGA, functional gait assessment; PVSQ, pediatric vestibular symptom questionnaire; PVID, pediatric visually induced dizziness questionnaire*.

## Discussion

The main findings were that children with concussion walked slower with eyes closed than controls, that gait speed with eyes open was not different between children with and without concussion, and that the GDT and GS-EC were equally able to discriminate between children with and without concussion. The GDT is an objective measure that assesses a person's balance ability by comparing walking time with eyes open and the walking time with eyes closed in a standardized walking task (12), providing a simple objective measure of the effect of eliminating visual input on a simple walking task. Maintaining balance is a complex task that involves the integration of three separate sensory systems: somatosensory, visual, and vestibular system. Normally, one can maintain balance with the removal of one sensory system, such as walking in a dark room. When there is damage or alteration in functioning of more than one postural control system, the effect on balance may be more evident. During the GDT, removing visual input forces the child to rely on vestibular and somatosensory inputs.

The GDT was able to distinguish between healthy subjects and participants with concussions, representing the accepted criterion-validity. This ability to distinguish those with a concussion is a cumulative addition to the measure's ability to differentiate between healthy people and those with vestibular hypofunction (12).

In this study, the average difference in GDT score between participants with a concussion and healthy controls was 2 s, which is less than the 6 s difference reported by 12 in persons with vestibular hypofunction. Howell et al. suggested that adding a cognitive component during walking tasks can demonstrate differences in the performance of the walking task between healthy and concussed adolescents. However, the GDT is utilizing a single-task testing approach (the elimination of vision), which may explain the small difference between the groups seen in this study (10, 12).

In addition to the GDT, walking with eyes closed and the FGA score were able to differentiate between healthy participants and participants with a concussion, whereas normal walking speed was not different between children with and without concussion. The GDT, walking with closed eyes, and the FGA include tasks that require the participant to close their eyes during walking, which forces a participant to rely more on the somatosensory and vestibular system, which may be affected because of the concussion injury (17). In contrast, normal walking was not able to differentiate between children with and without concussions. This may be due to the redundancy of sensory inputs that allow participants to rely on the visual and somatosensory systems when there is any reduction in vestibular inputs. Consistent with this finding, Brenker et al. found no differences in normal gait speed between adolescent athletes with and without concussion (8). Previous studies have reported that the vestibular system may be affected because of the concussive injury (18, 19).

Previous studies have compared differences in walking speed during various dual-task conditions with concussed and healthy adolescents (20, 21). Howell et al. (21) compared tandem walking speed with and without divided attention between youth athletes with and without and found that differences between groups were significant during the divided-attention-tandem walking but not during the undivided-attention-walking task. The findings of Howell et al. (21) were consistent with our findings, where there were differences in walking speed between groups walking with eyes closed. Previous studies showed that increasing the complexity of a functional task, such as normal gait speed, by adding a concurrent cognitive task or restricting the base of support (i.e., tandem walking), affects the performance of the task and enhances the tasks ability to discriminate between adolescents with and without concussion (20, 21).

Children with concussions who reported having dizziness demonstrated worse performance in all the functional tests in this study (i.e., GS, GS-EC, GDT, and FGA), while the presence of spinning sensation or migraine did not show differences in those functional tests. Consistent with this finding, Lue et al., in their study about the signs and symptoms that predict a protracted concussion recovery, found that between 12 different post-concussion signs and symptoms, only dizziness indicated a protracted recovery of concussion (22).

### Limitations

Although GDT was able to distinguish between children with and without concussion, a more useful validation of the GDT is to assess the ability of the GDT to distinguish between children with different diagnoses. Time since the concussion is an important factor in managing individuals with a concussion. Although concussed participants in this study were from a wide range of injury onset, they were recruited while they were seeking medical intervention for their concussion symptoms (23–25).

Another limitation was the difference in gender between the children with a concussion and the healthy children. Although we managed to have an equal number of male and female children in the control group, we recruited all available children with concussions, which resulted in imbalanced gender distribution. However, the one-way ANCOVA analysis was used to adjust for demographic differences between the groups, and neither gender, height, nor age affected the differences in the GDT scores.

## Conclusion

The GDT is a feasible, valid, and objective test to discriminate between children with and without concussions. It is a simple test that requires a stopwatch and a marked 20-ft hallway and can be performed within 1–2 min.

## Data availability statement

IRB approval must be obtained from the University of Pittsburgh to share patients' information. Requests to access the datasets should be directed at: Abdulaziz Alkathiry, a.alkathiry@mu.edu.sa.

## Ethics statement

The studies involving human participants were reviewed and approved by the institutional review board at the University of Pittsburgh, PA, USA. Written informed consent to participate in this study was provided by the participants' legal guardian/next of kin.

## Author contributions

Conceptualization and visualization: SW and AA. Data curation, methodology, validation, and writing: AA, SW, SA, and BA. Formal analysis: AA and SA. Project administration, software: AA. Resources and supervision: SW. All authors contributed to the article and approved the submitted version.

## Conflict of interest

The authors declare that the research was conducted in the absence of any commercial or financial relationships that could be construed as a potential conflict of interest.

## Publisher's note

All claims expressed in this article are solely those of the authors and do not necessarily represent those of their affiliated organizations, or those of the publisher, the editors and the reviewers. Any product that may be evaluated in this article, or claim that may be made by its manufacturer, is not guaranteed or endorsed by the publisher.
